# Exogenous Sphingosine-1-Phosphate Boosts Acclimatization in Rats Exposed to Acute Hypobaric Hypoxia: Assessment of Haematological and Metabolic Effects

**DOI:** 10.1371/journal.pone.0098025

**Published:** 2014-06-02

**Authors:** Sonam Chawla, Babita Rahar, Mrinalini Singh, Anju Bansal, Deepika Saraswat, Shweta Saxena

**Affiliations:** Experimental Biology Division, Defence Institute of Physiology and Allied Sciences, Defence Research and Development Organisation, Ministry of Defence, Lucknow Road, Timarpur, Delhi, India; VU University Medical Center, Netherlands

## Abstract

**Background:**

The physiological challenges posed by hypobaric hypoxia warrant exploration of pharmacological entities to improve acclimatization to hypoxia. The present study investigates the preclinical efficacy of sphingosine-1-phosphate (S1P) to improve acclimatization to simulated hypobaric hypoxia.

**Experimental Approach:**

Efficacy of intravenously administered S1P in improving haematological and metabolic acclimatization was evaluated in rats exposed to simulated acute hypobaric hypoxia (7620****m for 6 hours) following S1P pre-treatment for three days.

**Major Findings:**

Altitude exposure of the control rats caused systemic hypoxia, hypocapnia (plausible sign of hyperventilation) and respiratory alkalosis due to suboptimal renal compensation indicated by an overt alkaline pH of the mixed venous blood. This was associated with pronounced energy deficit in the hepatic tissue along with systemic oxidative stress and inflammation. S1P pre-treatment improved blood oxygen-carrying-capacity by increasing haemoglobin, haematocrit, and RBC count, probably as an outcome of hypoxia inducible factor-1α mediated erythropoiesis and renal S1P receptor 1 mediated haemoconcentation. The improved partial pressure of oxygen in the blood could further restore aerobic respiration and increase ATP content in the hepatic tissue of S1P treated animals. S1P could also protect the animals from hypoxia mediated oxidative stress and inflammation.

**Conclusion:**

The study findings highlight S1P’s merits as a preconditioning agent for improving acclimatization to acute hypobaric hypoxia exposure. The results may have long term clinical application for improving physiological acclimatization of subjects venturing into high altitude for occupational or recreational purposes.

## Introduction

Over five decades have passed since the ‘Silver Hut’ expedition and inception of ‘high altitude biology’ [1]. Despite the exhaustive scientific knowledge generated for the underlying mechanisms of biological responses to hypobaric hypoxia exposure, graded or staged ascent are the only established recommendations for improving acclimatisation. Though graded ascent facilitates gradual physiological compensations to set in the body which is beneficial for acclimatization, due to its time course, it cannot be a strategy for rapid acclimatization [2]. Pharmacological mitigation agents like acetazolamide and dexamethasone are successful as therapeutics against high altitude maladies rather than as “acclimatisation agents”. Moreover, they impose unacceptable adverse side effects and potentially compromise natural acclimatization process [3].

Hypoxic pre-conditioning, prior to the actual hypoxia exposure, initiates the physiological adjustments to suboptimal oxygen and it can be achieved by a brief exposure in hypobaric simulation chambers [4]. World Health Organization statistics estimate more than thirty five million people travelling annually to higher altitudes for recreational or military purposes across the globe [5]. An effective pharmacological agent for rapid acclimatisation is thus an urgent requirement, since accessibility of hypobaric chambers or normobaric hypoxic air exposure is limited in its use for masses. Pharmacological intervention with a hypoxia mimetic - hypoxia inducible factor-1α (HIF-1α) stabilizers, is an emerging concept to simulate the hypoxia induced adaptive molecular and physiological cascade of events leading to rapid acclimatization [6], [7]. An ideal pharmacological agent shall help in improving high altitude acclimatization while minimally interfering with the normal physiology, since rapid acclimatisation is an imperative mainly for soldiers, pilots and mountaineers for whom fitness is a prime.

Present study proposes sphingosine-1-phosphate (S1P), a blood borne lipid with an array of biological activities, as a potential preconditioning agent facilitating acclimatization to hypobaric hypoxia. S1P is produced by platelets, RBCs and the endothelium, and is maintained at a physiological concentration up to 1 µM in the plasma [8]. Its signalling functions are executed *via* five G-protein coupled receptors known till date – S1P Receptors (S1P_1–5_), and through potential intracellular targets yet unknown. Exogenous administration of S1P and its functional agonists have shown immense promise in the *in vivo* models of respiratory, cardiovascular, cerebral and renal disorders where underlying hypoxia is either a cause or an outcome [9]. In several pre-clinical investigations, exogenous S1P has proven to be an effective prophylaxis against ischaemia-reperfusion, and it, in fact, mimics the protection conferred by hypoxic pre-conditioning [10], [11]. These outcomes are largely linked to S1P mediated stabilization of hypoxia inducible factor -1α (HIF-1α), the master regulator of hypoxia adaptive gene expression [12]. Further, since S1P is synthesized within the body, its regulated modulation at systemic and tissue level may offer a safer prophylactic strategy against hypoxia mediated maladies compared to a chemical hypoxia mimetic.

The present study indicated that systemic pre-treatment of rats with exogenous S1P, within its physiological range, facilitated acclimatisation to hypobaric hypoxia primarily through enhanced blood oxygen carrying capacity, optimized hepatic bioenergetics and protection from oxidative stress and inflammation. We propose S1P mediated HIF-1α stabilization and renal S1P receptor 1 (S1P_1_) dependant haemo-concentration being the key underlying mechanisms for the observed efficacy. It is a pioneering study suggesting potential use of S1P as a prophylactic agent to facilitate rapid acclimatisation to high altitude.

## Methods and Materials

### Drugs, Chemicals Reagents and Other Materials

Enhanced Chemiluminisence kit, S1P and all other chemicals used were obtained from SIGMA, USA. ELISA kits for cytokines and C-RP were from BD OptEIA™, USA and Millipore, USA. ELISA kits for HIF-1α and VEGF were purchased from R & D Systems, USA. GLUC-PAP, serum iron and TIBC estimation kits were purchased from Randox Laboratories, USA. Antibodies for western blot were sourced from Santa Cruz, USA. iSTAT cartridges were purchased from Abbot, USA. ATP estimation kit was from Invitrogen, USA.

### Use of Animals and Ethics Statement

Thirty Sprague-Dawley rats (weight 180±20 g) were obtained from the institute’s animal house facility and were housed at an ambient temperature of 25±2°C, with a 12 hour diurnal cycle. Standard chow and sterile water was available to rats *ad libitum*. All protocols and experiments were approved by the institutional animal ethical committee and were in compliance with the Committee for the Purpose of Control and Supervision of Experiments on Animals (CPCSEA), India.

### Experimental Design

The rats were randomly divided into 5 groups – normoxia control, vehicle control (equivalent to hypoxia control), S1P dosed –1, 10 and 100 µg/kg body weight (µg/kg b.w.) with six animals in each group. S1P was prepared as 1 mM stock in 10 mM NaOH which was diluted to required concentrations using drug delivery vehicle (0.1% BSA in normal saline (pH 7.8)). The three doses of S1P were administered intravenously in separate groups of animals for three consecutive days. Thirty minutes following the last dose of S1P, animals were ascended to 7620 m at a rate of 304 m/min in an animal decompression chamber and thereafter they were maintained at 7620 m for 6 hours. Following the hypoxia exposure, the animals were descended to sea level at the rate of 304 m/min. Normoxia control animals were maintained in the same room where hypoxia group animals were exposed in decompression chamber to minimize the experimental errors due to environmental factors other than hypoxia.

### Blood and Tissue Collection

Following hypoxia exposure, rats were anaesthetized with intra-peritoneal injection of anaesthesia combination - ketamine (80 mg/kg b.w.) and xylazine (8 mg/kg b.w.). Retro-orbital blood samples were collected under deep anaesthesia for iSTAT analysis, plasma and serum separation. Heparin (10 IU/mL) was used as anticoagulant for blood samples for iSTAT analysis. K3-EDTA (1.5 mg/mL) was used as anticoagulant for blood samples for haematology, biochemical analysis and plasma separation. Following blood sample collection whole body vascular perfusion to remove residual blood from the tissues was done through left ventricle using 30 mL of ice cold normal saline with perfusion rate of 3 ml/minute using standard technique. Following perfusion, liver and kidneys were collected. All tissues, plasma and serum samples were snap frozen in liquid nitrogen and stored at −80°C till further studies.

### Blood Gas and Clinical Chemistry Analysis

i-Stat analyzer (Abbot, East Windsor, N.J., USA) was used for analysis of blood pH, blood gas composition (pCO_2_ - partial pressure of CO_2,_ pO_2_ - partial pressure of O_2,_ SvO_2_ - percentage saturation of oxygen in mixed venous blood) and clinical chemistry parameters (Base Excess, Lactate, HCO_3_^−^ - bicarbonate, Na^+^ - ionized sodium). Utmost care was taken to avoid blood hemolysis while sample drawing and loading for iSTAT analysis.

### Haematology, Serum Iron and Total Iron Binding Capacity

Blood cell counts, Hb content and Hct estimations were performed in the blood sample using MS-4 Autoanalyzer (Melet Schloesing Laboratories, France). Serum iron and TIBC were estimated using commercial kits according to the manufacturer’s guidelines.

### HIF-1α, VEGF and EPO Quantification

Homogenates of liver and kidney tissues were prepared in PBS (pH = 7.4), fortified with protease inhibitor cocktail to estimate HIF-1α accumulation using total HIF-1α kit. Erythropoietin (Epo) and Vascular Endothelial Growth Factor (VEGF) levels were estimated in plasma samples using commercial ELISA kits and following manufacturer’s guidelines.

### S1P Receptor 1 (S1P_1_) Expression in the Renal Tissue: Western Blot Analysis

Kidney tissue was homogenized in PBS (pH = 7.4) fortified with protease inhibitor cocktail to prepare a 10% homogenate. The protein sample for SDS-PAGE was prepared by mixing homogenate with 6X Laemelli buffer (0.25 M Tris-HCl pH 6.8, 10% SDS, 0.5% bromophenol blue, 0.5 M di-thiothretiol and 50% glycerol) and boiling the samples for 10 minutes. Fifty microgram protein was loaded and resolved on a 10% acrylamide gel. Resolved proteins were blotted on nitrocellulose membrane and blocked overnight in 3% BSA solution. Blocking solution and antibody dilutions were prepared in tris-buffered saline with 0.1% tween-20. Blot was incubated with anti-S1P_1_ (1∶1000) for 3 hours and then with anti-goat HRP labelled secondary antibody (1∶40,000) for 2 hours, at room temperature. Enhanced chemiluminisence detection kit was used to develop the blots and captured on X-ray film. Loading control used was α-tubulin (1∶1000). Densitometry analysis was done using ImageJ software.

### Energy Metabolism

20% homogenate of liver tissue was prepared in 0.154 M potassium chloride solution (pH = 7.5). CS [13], SDH [14], HK [15], LDH [16] enzyme activities were measured as markers of aerobic *vs.* anaerobic metabolism. Liver glycogen reserve was estimated according to Zhang P et al. [17]. Liver glucose was estimated using GLUC-PAP kit and liver Adenosine triphosphate (ATP) content was estimated using the ATP determination kit.

### Oxidative Stress Markers

ROS, GSH, GSSG were quantified in the whole blood haemolysate samples [18]–[20]. Plasma lipid peroxidation was determined by measurement of TBARS [21] and plasma protein carbonyl content was determined by 2,4-dinitrophenylhydrazine (DNPH) method [22].

### Inflammatory Markers

Pro-inflammatory cytokines *viz.* IFN-γ, TNF-α, IL-6, TGF-β, MCP-1 and anti-inflammatory cytokine IL-10 levels were estimated in the plasma sample using commercial kits. Plasma C-RP level was estimated using rat C-RP estimation kit. Arginase activity was measured in the whole blood haemolysate samples using manual method, briefly, samples were incubated with activation buffer (10 mM MnCl_2_ in 50 mM Tris-HCl, pH 7.5) to activate the arginase, and then with 0.5 M arginine (pH 9 7) at 37°C for 1 h. Reaction was terminated by addition of acid solution and the urea formation was detected using iso-nitroso-propiophenone solution, the absorbance was recorded at 540 nm [23].

### Data Analysis and Statistical Procedures

Reported data is from at least three experiments (performed in triplicates wherever relevant). All values are reported as mean ± standard deviation (SD). Drug groups were statistically compared to hypoxia and normoxia control groups using one-way ANOVA/*Post hoc* Bonferroni’s analysis method.

## Results

### S1P Pre-conditioning Enhances Blood Oxygen Carrying Capacity and Optimizes Renal Compensation to Counter Deleterious Effects of Acute Exposure to Hypobaric Hypoxia

Fall in the ambient partial pressure of oxygen due to simulated hypobaric hypoxia exposure caused a significant reduction in the mixed venous blood oxygen partial pressure (pO_2_) and oxygen saturation (SvO_2_) in the hypoxia control animals compared to the normoxic controls. In response to this fall, due to the hypoxia induced hyper-ventilatory response in these animals, blood pCO_2_ was reduced significantly (p<0.05) and thus pH was shifted toward alkalinity in the hypoxia controls. The most striking finding of this study was that S1P pre-conditioning could raise pO_2_ (p<0.05) and SvO_2_ (p≤0.01) in the mixed venous blood despite a marginal fall in the pCO_2_, 1 µg/kg b.w. S1P being the most effective dose (Table 1). Further, a significant fall in base deficit value was observed in hypoxia controls in comparison to normoxia controls (p<0.05), with a concomitantly decreased blood HCO_3_^−^ (p<0.05) and raised blood lactate level (p≤0.001). Interestingly, S1P pre-treatment, dose-dependently, restricted the fall in HCO_3_^−^ level and prevented lactate accumulation (p≤0.001 in comparison to hypoxia control), raising the base excess by nearly 50% in the 1 µg/kg b.w. group compared to their hypoxia counterparts. Blood Na^+^ level showed a fall following hypoxia exposure, though non-significantly in hypoxia control group, however a much robust fall in blood Na^+^ was observed in the S1P treated groups especially in the 1 µg/kg b.w. dose group (p<0.05).

**Table 1:** Effect of S1P on blood gases variables, lactate and electrolyte level in acute hypobaric hypoxia exposed rat.

Blood gas variables	Normoxiccontrol	Hypoxic control	S1P (1 µg/kg)+Hypoxia	S1P (10 µg/kg)+Hypoxia	S1P (100 µg/kg)+Hypoxia
**pH**	7.37±0.08	7.47±0.12	7.39±0.09	7.40±0.11	7.46±0.13
**pCO_2_** **[mmHg]**	58.20±8.88	37.93±5.65*	44.78±3.50	40.00±5.92*	37.47±8.94*
**pO_2_** **[mmHg]**	40.60±4.32	31.67±3.09*	43.00±3.54†	38.50±6.02	38.00±7.87
**Base Excess** **[mmol/L]**	−1.25±2.68	–6.33±1.25*	−3.25±1.48	−7.00±3.39*	−7.00±3.08*
**SvO_2_** **[%]**	70.40±8.40	55.67±7.76*	78.00±1.41††	72.25±7.01†	70.50±6.18†
**Lactate** **[mmol/L]**	0.97±0.24	2.36±0.27***	1.13±0.18†††	1.33±0.52††	1.47±0.07†††
**HCO_3_^−^** **[mmol/L]**	25.66±1.49	19.97±0.58**	23.00±1.50†	20.30±3.70	19.35±1.19
**Na^+^** **[mmol/L]**	155.60±2.65	150.00±8.49	144.00±5.24†	147.25±8.14	152.00±6.75

Values are means ± SD (*n* = **6**).

*p<0.05 compared with the normoxic control,

**p≤0.01 compared with the normoxic control,

***p≤0.001 compared with the normoxic control,

† *p*<0.05 compared with the hypoxic control,

†† *p*≤0.01 compared with the hypoxic control,

††† *p*≤0.001 compared with the hypoxic control.

Hypoxic exposure stimulated TIBC and serum iron level to support the hypoxia induced erythropoiesis (Table 2). S1P pre-treatment could further raise the baseline serum iron and TIBC significantly above the hypoxia control. Serum iron and TIBC levels were 468±82.50 µg/dl and 1.14±0.13 µg/dl, in the hypoxia control group while 1 µg/kg b.w. S1P treated group values were 644.11±81.94 µg/dl and 1.49±0.17 µg/dl, respectively. Haematological analysis indicated that pre-treatment with S1P at the dose of 1 µg/kg b.w. significantly increased RBC numbers (p≤0.01) and haemoglobin concentration (p<0.05). The raised RBC numbers following S1P pre-treatment led to a significantly higher haematocrit in comparison to the hypoxic control (p<0.05) (Table 2).

**Table 2:** Effect of S1P on oxygen-carrying-capacity in acute hypobaric hypoxia exposed rats.

Oxygen carrying capacity indicators	Normoxic control	Hypoxic control	S1P (1 µg/kg)+Hypoxia	S1P (10 µg/kg+Hypoxia	S1P (100 µg/kg)+Hypoxia
**Serum Iron (µg/dl serum)**	370.33±50.73	468.00±82.5	644.11±81.94** †	554.67±80.14**	410.50±81.44
**TIBC** **(µg/dl serum)**	0.749±0.133	1.14±0.13*	1.49±0.17*** †	1.28±0.11**	1.07±0.12*
**Haemoglobin (g/dl blood)**	11.60±0.40	13.55±1.29	15.78±0.55*** †	13.08±1.32	14.44±2.28
**RBC (Million/mm^3^)**	5.75±0.22	6.31±0.33	7.33±0.31*** ††	6.28±0.87	6.83±0.96
**Hematocrit** **(%)**	40.35±4.95	44.40±1.43	52.68±2.49** †	41.32±4.67	45.56±7.05

Values are means ± SD (*n* = **6**).

*p<0.05 compared with the normoxic control,

**p≤0.01 compared with the normoxic control,

***p≤0.001 compared with the normoxic control,

† *p*<0.05 compared with the hypoxic control,

†† *p*≤0.01 compared with the hypoxic control,

††† *p*≤0.001 compared with the hypoxic control.

### S1P Stabilizes HIF-1α and Boosts HIF-1α Mediated Hypoxia Adaptive Responses

**Figure 1:**
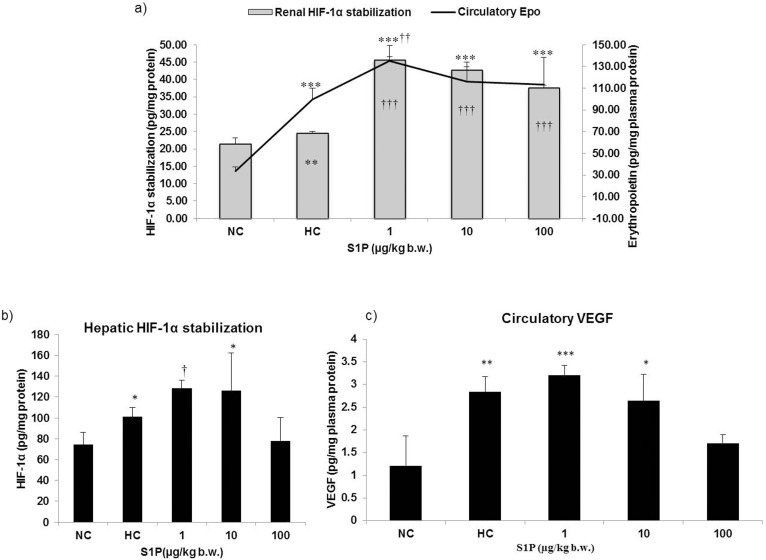
Effect of S1P treatment on HIF-1α accumulation and downstream gene expression. a) Renal HIF-1α accumulation and Epo accumulation in plasma. HIF-1α accumulation in the renal tissue homogenate and build-up of erythropoietin in plasma was quantified. b) Hepatic HIF-1α accumulation. c) Effect S1P pre-treatment on circulatory VEGF. Vascular endothelial growth factor (VEGF) was quantified in plasma of experimental animals. These estimations were carried out using sandwich ELISA, and were carried out in triplicates for each experimental animal. Values are representative of mean ± SD (n = 6). Statistical significance was calculated using ANOVA/*post hoc* Bonferroni. NC: Normoxia control, HC: Hypoxia control, 1: 1 µg S1P/kg b.w., 10: 10 µg S1P/kg b.w., 100: 100 µg S1P/kg b.w., *: p<0.05 compared with the normoxic control, **: p≤0.01 compared with the normoxic control, ***: p≤0.001 compared with the normoxic control, †: p<0.05 compared with the hypoxic control, ††: p≤0.01 compared with the hypoxic control, †††: p≤0.001 compared with the hypoxic control.

S1P pre-conditioning led to 1.9 fold higher HIF-1α level in the kidney tissue (p≤0.001) and 1.3 fold higher HIF-1α level in the liver tissue (p≤0.001) in 1 µg/kg b.w. S1P group than in hypoxia control group. However, the hypoxia control group also had 1.3 folds higher HIF-1α levels in both liver and kidney tissues than in normoxia control groups, indicating a non-hypoxic boost of HIF-1α in S1P treated animals (Figure 1a and b). Further, plasma Epo levels were also observed to be significantly higher following S1P pre-treatment compared to the hypoxia control groups (p<0.05) (Figure 1a). Epo being primarily secreted by the kidneys and its expression being under regulation of HIF-1α, the raised plasma Epo level could be attributed to higher HIF-1α level in the kidney. Further, VEGF is a pro-angiogenic mediator that leads to improved oxygen delivery to the hypoxic tissues and its expression is under regulation of HIF-1α. The difference in VEGF levels between normoxia *vs.* S1P treatment group (1ug/kg dose) was more statistically significant in comparison to the difference in VEGF levels between normoxia *vs.* hypoxia group. A marginal boost in plasma VEGF level was observed following S1P supplementation (at least in 1ug/kg S1P dose) in comparison to hypoxia control group (1c).

### S1P Modulates S1P_1_ Expression Pattern in Renal Tissue

**Figure 2:**
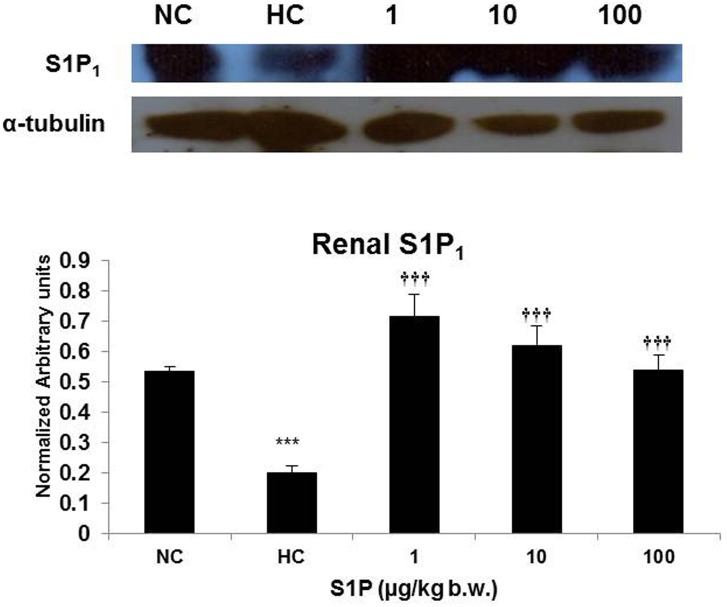
Effect of S1P treatment on S1P_1_ expression in renal tissue. Representative immune-blot of S1P1. Densitometric analysis of blot normalized against the loading control (α-tubulin). Values are representative of mean ± SD (n = 6). Statistical significance was calculated using ANOVA/*post hoc* Bonferroni. NC: Normoxia control, HC: Hypoxia control, 1: 1 µg S1P/kg b.w., 10: 10 µg S1P/kg b.w., 100: 100 µg S1P/kg b.w., *: p<0.05 compared with the normoxic control, **: p≤0.01 compared with the normoxic control, ***: p≤0.001 compared with the normoxic control, †: p<0.05 compared with the hypoxic control, ††: p≤0.01 compared with the hypoxic control, †††: p≤0.001 compared with the hypoxic control.

To detect the modulation of S1P_1_ expression induced by S1P at the protein level, we used western blot analysis followed by densitometric analysis of the immunoblot. Results indicate that S1P pre-treatment could boost the expression of S1P_1_ in renal tissue with maximum effect at 1 µg/kg b.w. S1P (Figure 2).

### S1P Facilitates Recovery from Hypoxia Associated Energy Deficit in Hepatic Tissue

**Figure 3:**
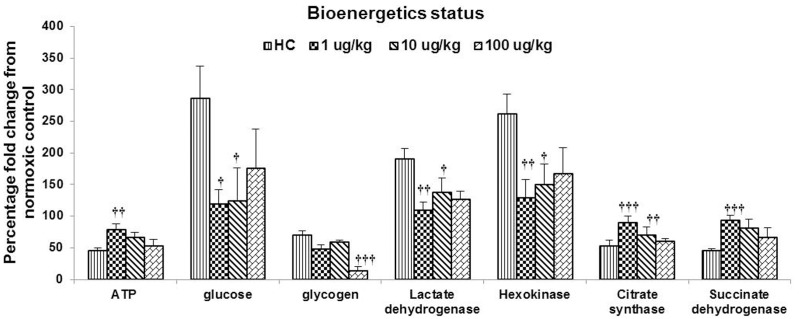
Effect of S1P treatment on bioenergetics status in hepatic tissue. Homogenate of hepatic tissue was analyzed for ATP content, tissue glucose, glycogen reserve, indicator of anaerobic metabolism (Lactate Dehydrogenase), glycolysis (Hexokinase) and citric acid cycle (Succinate dehydrogenase and Citrate Synthase). Each assay was carried out for each experimental animal thrice. Data is represented as mean percentage fold change against normoxic control. Values are representative of mean ± SD (n = 6). Statistical significance was calculated using ANOVA/*post hoc* Bonferroni. NC: Normoxia control, HC: Hypoxia control, 1: 1 µg S1P/kg b.w., 10: 10 µg S1P/kg b.w., 100: 100 µg S1P/kg b.w., *: p<0.05 compared with the normoxic control, **: p≤0.01 compared with the normoxic control, ***: p≤0.001 compared with the normoxic control, †: p<0.05 compared with the hypoxic control, ††: p≤0.01 compared with the hypoxic control, †††: p≤0.001 compared with the hypoxic control.

The sub-optimal oxygen availability during acute exposure to hypobaric hypoxia hampers with the aerobic metabolic pathways. As evident from Figure 3 and Table 1, the hypoxic control group switched to anaerobic glycolysis for energy production, indicated by a nearly 2 fold increase in the HK and LDH activities in the liver tissue and blood lactate accumulation. Citric acid cycle enzyme activities - CS and SDH, were depreciated by 50%. An overall energy deficit of 65% was detected in the hepatic tissue of the hypoxic control. Moreover, nearly 30% glycogen reserve was mobilized from the liver, with a concomitant high accumulation of glucose in the hepatic tissues.

S1P pre-treatment, dose-dependently, rescued the animals from the hypoxia associated energy deficit of the liver tissue, most significantly in the 1 µg/kg b.w. dose group where the tissue ATP content was compromised by a mere 20% (p≤0.01). Further a significant shift towards aerobic metabolism was indicated by a reigned HK (p≤0.01), LDH activity (p≤0.01) and blood lactate (p≤0.001), but raised CS (p≤0.001) and SDH (p≤0.001) activities, which were reasonably close to normoxic state. The depletion of glycogen reserve was higher in S1P treated group than that in hypoxic control, but the tissue glucose accumulation was only 19% more than the normoxia group.

### Exogenous S1P Alleviates Hypobaric Hypoxia Associated Oxidative Stress and Inflammation

Acute exposure to hypobaric hypoxia ensued oxidative and inflammatory stress in the hypoxia control group. A two-fold higher reactive oxygen species (ROS) generation (p≤0.01) in the blood cells led to ROS induced plasma lipid peroxidation (p≤0.01) and protein oxidation (p<0.05) (Table 3). Pre-treatment with S1P, most significantly at 1 µg/kg b.w. dose, alleviated the oxidative injury in the blood components as indicated by a significant fall in the TBARS (p<0.05) and protein carbonylation products (p≤0.01), as compared to the hypoxia control group. This could be an outcome of the observed significantly reigned ROS generation (2 fold fall, p<0.05) and raised GSH: GSSG ratio (p≤0.001), against the hypoxic control group.

**Table 3:** Effect of S1P on oxidative stress markers and arginase activity in acute hypobaric hypoxia exposed rats.

Blood Biochemical Indicators	Normoxiccontrol	Hypoxic control	S1P (1 µg/kg)+Hypoxia	S1P (10 µg/kg)+Hypoxia	S1P (100 µg/kg)+Hypoxia
**GSH/GSSG (Ratio)**	11.5±0.93	11.2±1.08	23.2±4.52*** †††	14.2±4.66	16.0±1.93*** †††
**ROS** **(RFU/g Hb)**	402.9±92.8	878.1±246.9**	435.0±140.5†	624.7±223.0	750.0±257.8
**TBARS** **(nM/mg protein)**	0.04±0.01	0.08±0.01**	0.05±0.01†††	0.04±0.01†	0.05±0.01†
**Protein carbonylation (µM/mg protein)**	1.11±0.33	2.01±0.43**	0.95±0.21††	0.98±0.20	1.05±0.32
**Arginase (Units/g Hb)**	119.5±12.87	132.9±13.37	86.5±14.98* ††	97.86±27.71††	141.94±28.97

Values are means ± SD (*n* = **6**).

*p<0.05 compared with the normoxic control,

**p≤0.01 compared with the normoxic control,

***p≤0.001 compared with the normoxic control,

† *p*<0.05 compared with the hypoxic control,

†† *p*≤0.01 compared with the hypoxic control,

††† *p*≤0.001 compared with the hypoxic control.

**Figure 4:**
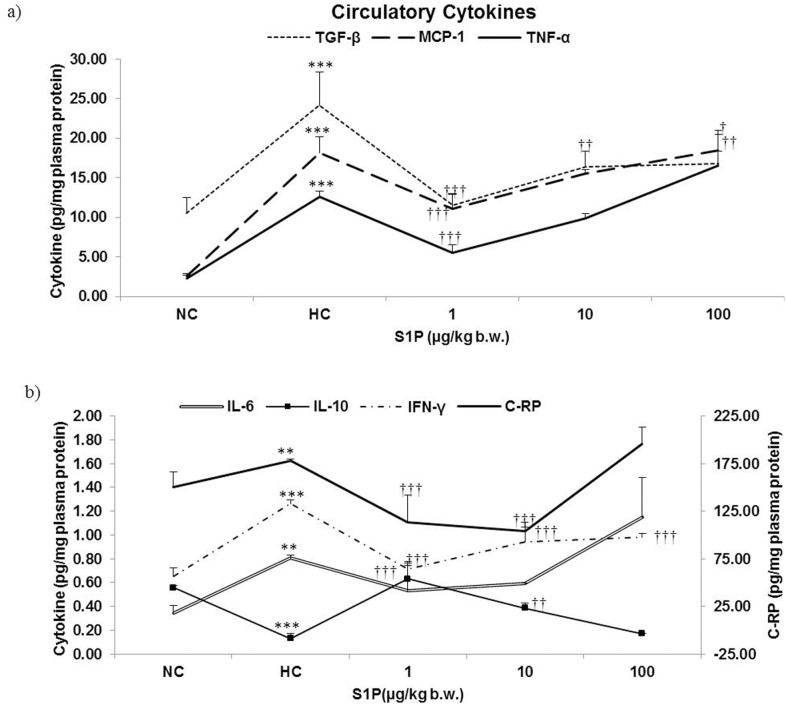
Effect of S1P treatment on circulatory pro- and anti-inflammatory markers. Markers of inflammation – IFN-γ, IL-6, TNF-α, MCP-1, TGF-β, C-Reactive protein (C-RP) and anti-inflammatory cytokine IL-10 were quantified post-exposure in plasma of experimental animals using sandwich ELISA, each animal represented in triplicate. Figure 4 a) describes TGF-β, TNF-α, MCP-1 Figure 4 b) describes IL-6, IFN-γ, C-RP, IL-10 levels in the systemic circulation. Values are representative of mean ± SD (n = 6). Statistical significance was calculated using ANOVA/*post hoc* Bonferroni. NC: Normoxia control, HC: Hypoxia control, 1: 1 µg S1P/kg b.w., 10: 10 µg S1P/kg b.w., 100: 100 µg S1P/kg b.w., *: p<0.05 compared with the normoxic control, **: p≤0.01 compared with the normoxic control, ***: p≤0.001 compared with the normoxic control,†: p<0.05 compared with the hypoxic control, ††: p≤ 0.01 compared with the hypoxic control, †††: p≤ 0.001 compared with the hypoxic control.

Further, the inflammatory stress as an outcome of enhanced pro-inflammatory cytokine response (p≤0.001) was reigned by significant fall in the plasma IL-6, IFN-γ, TNF-α, MCP-1, TGF-β and C-RP (p≤0.001), and a raised anti-inflammatory IL-10 levels (p≤0.001) following S1P pre-treatment (Figure 4a and b).

It is well known that anti-inflammatory marker, nitric oxide (NO) production, depends on the competitive availability of l-arginine to nitric oxide synthase (NOS) or arginase enzyme. Thus, in view of this, we estimated the arginase activity in the blood haemolysate samples. Interestingly, S1P pre-treatment at the dose of 1 µg/kg b.w. significantly reduced the arginase activity in comparison to hypoxia control (p≤0.001) as well as normoxia controls (p≤0.001) (Table 3).

## Discussion

The present study proposes that pre-treatment of rats with exogenous S1P boosts their acclimatization response *via* improvement of blood oxygen carrying potential, compensatory molecular and haematological responses and restoration of aerobic energy generation. The preclinical screening model used in the present study was a rat model of acute exposure to hypobaric hypoxia that not only showed signs of severe oxidative stress and inflammation but also mimicked development of acute molecular and physiological responses observed in human subjects exposed to hypobaric hypoxia.

Hyper-ventilation is the foremost physiological response to hypoxia exposure to maintain optimal alveolar pO_2_ which in turn drives the alveolar pCO_2_ to fall below physiological threshold resulting in systemic hypocapnia. This hypocapnia further invokes a compensatory respiratory alkalosis to restore fall in blood pH [24]. In the present study, the blood gas composition of the hypoxia control rats recapitulated the above phenomenon following exposure to hypobaric hypoxia. As indicated in table 1, a significantly reduced pCO_2_ level along with raised pH of the mixed venous blood in hypoxia control rats was observed indicating hyperventilation mediated respiratory alkalosis. However, a parallel reduction in blood HCO^3−^ level points at an incomplete compensatory metabolic acidosis being operational as well. The base deficit due to excessive loss of HCO^3−^ by the kidneys and acid excess due to anaerobic respiration induced lactic acidosis led to an overtly negative base excess value in hypoxia control group [25]. The lactic acid accumulation is an indicator of anaerobic respiration in the hypoxia control rats due to alarmingly reduced pO_2_ level and oxygen saturation in mixed venous blood (SvO_2_) in these animals, which hints at exhaustion of oxygen extraction from the blood, throwing off balance the oxygen delivery and consumption [26]. Whereas, in the S1P treated animals pCO_2_ levels were only moderately reduced in dose dependent manner following hypoxia exposure and so was the compensatory HCO^3−^ loss by the kidneys. This compensatory metabolic acidosis, at least in animals treated with 1 µg/kg S1P, appears to be enough to maintain near physiological blood pH (Table 1). Another interesting observation was that even with this moderate fall in pCO_2_ level in the 1 µg/kg S1P supplemented animals, the pO_2_ level as well as SvO_2_ values were significantly higher (Table 1). Though we agree that these are the trends in mixed venous blood but a higher value of oxygen in mixed venous blood accompanied with a significantly reduced level of blood lactate in S1P supplemented rats certainly indicates at improved oxygen transport to the tissues leading to controlled anaerobic respiration (Table 1).

We propose that the S1P induced improvement in oxygen carrying potential observed in the present study could be due to three factors, firstly, haemo-concentration due to enhanced diuresis in these animals as indicated by a pronounced dose dependent fall in blood Na^+^ level in S1P treated animals in comparison to hypoxia controls (Table 1), second, a high hematocrit, RBC and haemoglobin content due to pre-existing higher erythropoietin level under the influence of S1P (Table 2; Figure 1a) and third, improved iron binding capacity indicated by serum iron and TIBC (Table 2). Recently, Zhu et al. have illustrated diuretic properties of S1P and have suggested it to be an important regulator of sodium homeostasis *via* S1P_1_ in the renal medulla [27]. In the present study, we observed S1P_1_ up-regulation in the renal tissue following S1P pre-treatment especially in the 1 µg/kg b.w. group which coexisted with haemo-concentration and significantly reduced blood Na^+^ level as well, further endorsing potential diuretic and natriuretic properties of S1P (Table 1 and Figure 2). Preconditioning the animals with S1P for three days prior to hypoxia exposure led to increased HIF-1α level (Figure 1a) which further caused higher levels of plasma Epo (Figure 1a) [28]. The kidney is highly sensitive to oxygen levels and plays a central role in mediating the hypoxic induction of RBCs *via* Epo synthesis, a key step for physiological adaptation to sub-optimal oxygen [29]. Increased Epo, within 2 hours of hypobaric hypoxia exposure, triggers erythropoiesis over days, which is an adaptive response to facilitate acclimatization to high altitude [30]. In the present study, a higher plasma Epo level in S1P treated animals prior to hypoxia exposure might have contributed to improved oxygen-carrying capacity, at least partially. Further, considering the fact that erythroid marrow consumes more than 70–80% of plasma iron, it is conceivable that a parallel increase in serum iron transport capacity would facilitate Epo-mediated erythropoiesis [31]. It was observed that pre-conditioning with S1P could maintain relatively higher serum iron and TIBC (Table 2), an index of transferrin content, which would have facilitated at least initiation of erythropoiesis prior to hypoxia exposure in these animals. The increase in serum iron and TIBC could be a manifestation of higher HIF-1α content since it is known to up regulate the expression of transferrin protein and its receptors [32].

Hypoxia inhibits enzymes participating in the electron transport chain such as NADH-dehydrogenase and Cytochrome-C-oxidase [33]. Since NADH is the rate limiting factor of isocitrate dehydrogenase, α-ketoglutarate dehydrogenase, succinate dehydrogenase, hexokinase and pyruvate dehydrogenase, the accumulation of NADH leads to feedback repression on the activities of these enzymes [34]. In the present study, up regulation of HK activity with a concomitant down regulation of SDH and CS activities in the liver of hypoxia control rats could be a part of early acclimatization adjustments to facilitate operation at severely suppressed ATP turnover rate during hypoxia. As ATP generation by oxidative phosphorylation in these animals begins to fall off, the energy deficit was made up by activation of anaerobic ATP supply pathways, as indicated by increase in liver LDH activity and blood lactate level. The elevated lactate production could also be attributed to increased rates of glycogenolysis, which is a known effect of hypoxia exposure [35], however extremely high liver glucose suggests poor glucose utilization in hypoxia control rats. It is noteworthy that the improved oxygen transport potential in S1P supplemented animals led to better glucose utilization towards maintenance of ATP turnover *via* oxidative phosphorylation with hexokinase, succinate dehydrogenase and citrate synthase activities close to the levels in normoxic animals (Figure 3). There was less dependence on anaerobic ATP generation as indicated by reduced LDH activity and lactate levels in these animals (Figure 3, Table 1).

**Figure 5:**
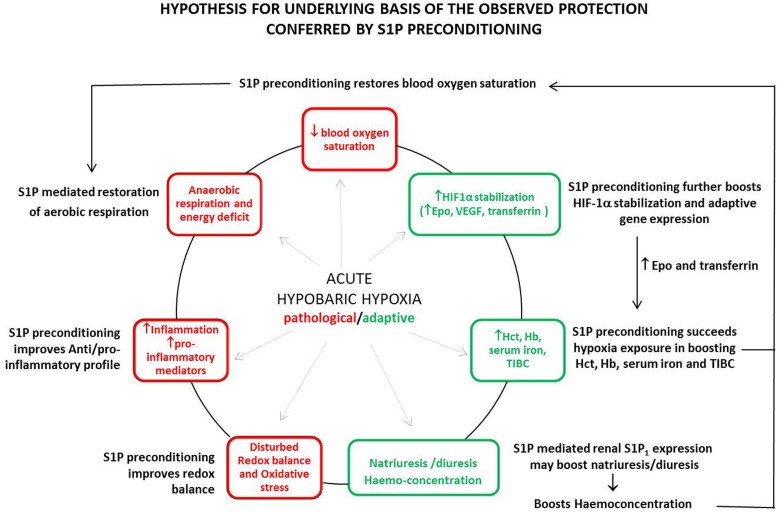
Hypothesis for underlying basis of the observed protection conferred by S1P preconditioning. Exposure to hypobaric hypoxia evokes pathological (red boxes in circle) as well as adaptive (green boxes in circle) responses in the body, as an outcome of compromised systemic oxygen bioavailability. The strength of adaptive responses in unacclimatized individuals is insufficient to confer protection, arising the need for pharmacological mitigation. The study reports sphingosine-1-phosphate mediated preconditioning (black text outside the circle) to potentially confer protection against pathological milieu as well as boost the adaptive responses. Sphingosine-1-phosphate mediated boost in haemoglobin, haematocrit, RBC count, serum iron, TIBC, haemo-concentration and oxygen bioavailability culminates into successful acclimatization.

The concept that hypoxia can induce inflammation has gained general acceptance [36]. Rats when exposed to hypoxia secreted high levels of pro-inflammatory cytokines *viz.* TNF-α, IL-6, IFN-γ, TGF-β, MCP-1 and C-RP (Figure 4a and b). Preconditioning with exogenous S1P restored the balance between anti-inflammatory and pro-inflammatory cytokines with significantly high levels of anti-inflammatory IL-10 levels (Figure 4b). There are very few studies reporting S1P’s potential to favour a shift towards anti-inflammatory responses by inhibiting TNF-α, IL-12 and increasing IL-10 production in lymphocytes [37]–[39]. HIF-1α stabilization has been attributed to propagate anti-inflammatory responses while down-regulating pro-inflammatory responses. In the light of existing evidences, it may be inferred that S1P mediated HIF-1α accumulation could have modulated cytokine expression which resulted in a pronounced anti-inflammatory outcome [40]. Apart from cytokines, S1P also reduced other pro-inflammatory markers like plasma arginase activity and C-RP level (Figure 4). The nitric oxide (NO) production has been shown to be altered in hypoxia and its regulation is crucial in the development of hypertensive states induced by hypoxia [41], [42]. Reduction of arginase activity, an indirect indicator of nitric oxide synthase (NOS) activity, following S1P supplementation appears to be associated with pronounced hypoxia tolerance in these rats (Table 3). The over-expression of arginase has emerged as a hypoxia susceptibility marker [43] and the present finding further strengthen this hypothesis. On the other hand, C-RP is an acute phase protein produced primarily from the liver and is stimulated by the release of cytokines, such as IL-6 [44]. As a proof of anti-inflammatory properties of S1P, the present study clearly indicates that a fall in IL-6 following pre-treatment with S1P prior to hypoxia exposure led to a concomitant fall in plasma C-RP level (Figure 4). Further, our data clearly demonstrates that S1P preconditioning also protects the animals against hypoxia induced oxidative stress (Table 3). An increased GSH/GSSG ratio and significantly reduced ROS generation and associated oxidative damage of cellular lipid and proteins indicate at anti-oxidant properties of S1P. All these observations are perhaps incredibly relevant for pre-conditioning potential of S1P since occurrence of oxidative stress and inflammation in response to hypoxia is clinically relevant [45]. In light of the findings of the present study, we propose that S1P mediated preconditioning could dampen most of the known hypoxia mediated ill-effects such as lower oxygen saturation, energy deficit, inflammation and oxidative stress while boosting the protective responses such as haemo-concentration and HIF-1α mediated adaptive gene expression, potentially culminating into successful acclimatization (Figure 5).

## Conclusion

Our study has demonstrated that key mechanisms underlying the pre-conditioning benefits of systemic S1P, at least 1 µg/kg b.w. dose, include HIF-1α accumulation, haematological and hepatic bio-energetic adaptation, anti-oxidative and anti-inflammatory properties. This study also paves the way for future pre-clinical studies to explore pharmacological efficacy of systemic S1P administration against exposure to sub-chronic and chronic hypobaric hypoxia to promote clinical utility of this bioactive lipid.
